# Dynamic Anemia Status from Infancy to Preschool-Age: Evidence from Rural China

**DOI:** 10.3390/ijerph16152761

**Published:** 2019-08-02

**Authors:** Lei Wang, Mengjie Li, Sarah-Eve Dill, Yiwei Hu, Scott Rozelle

**Affiliations:** 1 International Business School, Shaanxi Normal University, Xi’an 710119, China; 2 Rural Education Action Project, Stanford University, Stanford, CA 94305, USA; 3 School of Physics and Optoelectronic Engineering of Xidian University, Xi’an 710126, China

**Keywords:** dynamic anemia status, anemia, early childhood, longitudinal study

## Abstract

Anemia is a serious nutritional deficiency among infants and toddlers in rural China. However, it is unclear how the anemia status changes among China’s rural children as they age. This study investigates the prevalence of anemia as children grow from infancy to preschool-age, as well as the dynamic anemia status of children over time. We conducted longitudinal surveys of 1170 children in the Qinba Mountain Area of China in 2013, 2015 and 2017. The results show that 51% of children were anemic in infancy (6–12 months), 24% in toddlerhood (22–30 months) and 19% at preschool-age (49–65 months). An even larger share of children (67%) suffered from anemia at some point over the course of study. The data also show that although only 4% of children were persistently anemic from infancy to preschool-age, 8% of children saw their anemia status deteriorate. We further found that children may be at greater risk for developing anemia, or for having persistent anemia, during the period between toddlerhood and preschool-age. Combined with the finding that children with improving anemia status showed higher cognition than persistently anemic children, there is an urgent need for effective nutritional interventions to combat anemia as children grow, especially between toddlerhood and preschool age.

## 1. Introduction

As one of the most vulnerable groups in the world, children are disproportionately at risk for health and nutrition issues [1]. One of the most common nutritional deficiency diseases among children is anemia. The World Health Organization (WHO) estimates that anemia affects 293 million children under 5 years old, accounting for 47.4% of children in the same age group globally [2]. The majority of these children are concentrated in developing countries [3].

International studies have shown that anemia during early childhood can lead to consequences in both the short and long term. In the short term, children with anemia have significantly lower mental, motor and socioemotional development than those without anemia [4,5,6,7]. Moderate and severe anemia are also associated with increased child morbidity and mortality [8]. In the long term, childhood anemia has long-lasting effects on cognitive ability, even after controlling for social background variables, gender and birth weight [9]. In part due to these cognitive delays, childhood anemia also has been linked to poor school performance, lower educational attainment and worse employment outcomes in adulthood [9,10,11,12].

In China, despite overall improvements in child health over the last three decades, anemia remains a serious problem among rural infants (0–1 years) and toddlers (1–3 years). Large-scale studies of rural infants in China have found rates of anemia between 42% and 49% [13,14,15], which exceed the 40% threshold set by the WHO for consideration as a “severe public health problem” [16]. Additionally, although international studies have found a natural downward trend in anemia rates as children grow older [17,18,19,20], the prevalence of anemia remains high among China’s rural toddlers. A study of China’s major rural sub-populations, which altogether represent 69% of rural families in China, found that 30% of toddlers aged 18–30 months suffered from anemia [21]. Another nationally-representative study found that 32% of toddlers aged 12–23 months and 18% of toddlers aged 24–35 months in poor rural areas suffered from anemia [13].

While there is comparatively little research on anemia among children of preschool age (3–5 years), the existing literature suggests that rates of anemia are still high among rural China’s preschool-aged children [22,23]. A study of nearly 10,000 rural children across 7 provinces conducted from 1985 to 1989 found that 28% of children aged 3–5 years were anemic [23]. A more recent study conducted in rural Yunnan Province in 2004 found around 32% of 3 to 5-year-old children to be anemic [22]. However, there are few studies of anemia among preschool-aged children in rural China, and the existing studies are relatively outdated. Considering the high prevalence of anemia among China’s rural infants and toddlers documented in the recent literature, there is a need for studies measuring the prevalence of anemia among preschool-age children in rural China.

Little is also known about the dynamic anemia status of children in developing settings such as that of rural China. Dynamic anemia status refers to changes in a child’s anemia status as they age. For example, some children may be persistently anemic or never anemic, while others may see their anemia status improve or deteriorate over time. Studies in the United States have found that changes in the anemia status of young children are common. A longitudinal study of children aged 6–59 months found that, while only 1.7% of children were persistently anemic over the course of the study, 16.4% of initially non-anemic children became anemic within 2 years [24]. Another longitudinal study of low-income children aged 12–36 months in the United States found that after one year, 30% of initially anemic children remained anemic, while 10% of non-anemic children became anemic [25]. However, to our knowledge, no studies have yet examined the dynamic anemia status of children in developing countries and regions, where childhood anemia is most prevalent.

Existing international research also suggests that differences in dynamic anemia status may lead to differences in the cognitive and non-cognitive development of young children. A study of infants aged 9–12 months in Chile found that persistent anemia for three or more months was linked to lower motor and cognitive scores, and the authors of this research concluded that if anemic infants were to continue with iron-deficient diets, their psychomotor performance would further deteriorate [26]. However, this study focused only on the persistence of anemia in infancy and did not examine childhood anemia in toddlerhood or preschool-age, or how changes (improvement or deterioration) in a child’s anemia status from infancy to preschool-age may affect cognitive development. In fact, to date, no study has analyzed the links between dynamic anemia status and any measures of early childhood development.

If changes in a child’s anemia status are indeed associated with better or worse developmental outcomes, what factors are linked with these changes? Studies from the United States have found that children of color are more likely to experience persistent anemia [25], and that children who had been breastfed for at least 25 weeks were more likely to see their anemia status improve [27]. However, no studies have examined the risk factors for changes in anemia status in the context of developing settings.

Additionally, although there is no research linking socioeconomic status to dynamic anemia status, existing studies show that early childhood anemia is associated with factors reflecting poor socioeconomic circumstances. Low family income, low parental education levels, young motherhood, and large family size have all been linked to higher rates of early childhood anemia [28,29,30,31,32,33]. Studies have also found low birth weight to be a strong predictor of anemia in childhood [34,35,36]. It is reasonable to consider whether these factors may also be associated with certain changes in anemia status, as they may provide specific indicators to target children who are more vulnerable to persistent anemia or deterioration in anemia status. This information may also help researchers and policymakers to improve interventions aimed at reducing the prevalence of childhood anemia.

The overall goal of this paper is to understand the dynamic anemia status of children in poor rural areas of China as they grow from infancy to preschool-age. In pursuit of this overall goal, we have four specific objectives. First, we describe the prevalence of anemia among sample children in infancy, toddlerhood and preschool-age, respectively. Next, we describe the changes in anemia status as children grow from infancy to preschool-age. Third, we compare the developmental outcomes of children who are persistently anemic, children who develop anemia over time, children who recover from anemia over time, and children who never experience anemia. Finally, we identify individual and household factors that may be correlated with each category of dynamic anemia status.

The remainder of this paper is structured as follows. The next section presents our methods, including sample selection, data collection and statistical methods. Section 3 presents our results. Section 4 discusses the results and concludes the paper.

## 2. Methods

### 2.1. Ethical Review

Approval for all data collection activities were obtained from the Stanford University Institutional Review Board (Protocol ID 25734), and from the Sichuan University Ethical Review Board (Protocol ID 2013005-01). We also obtained oral consent from all participating caregivers for their own and their infants’ involvement in the study. All participants were aware of the risks involved and understood that their participation was purely voluntary. Children who were found to have severe anemia were referred to the local hospital for treatment.

### 2.2. Sample Selection

The data presented in this paper come from a longitudinal study of children and households in 11 counties located in the Qinba Mountain Area of China. Although this region is recognized by China’s central government as a concentrated area of poverty, incomes in this area have increased significantly in recent years due to government-led poverty alleviation efforts [37]. Therefore, while this region is relatively poor, it is not one of the poorest in rural China [38].

The study sample was selected in 2013 using a three-step sampling protocol. First, all townships in the 11 sample counties were included in the study sample, with two exceptions: the research team excluded all townships located in county seats, as well as townships that did not contain any villages with a population of 800 or more. After applying the two exclusion criteria, 174 townships were included in the sample. Next, two sample villages in each township were randomly selected for inclusion in our study, totaling 351 villages. Finally, in each sample village, all registered newborns within the target age range (6–12 months) were enrolled in the study. In total, the baseline sample included 1802 infants aged 6–12 months living in 351 villages in 11 counties (for more detailed information on sample selection, please refer to [14,39]).

In 2015, two years after the initial sample selection, the research team conducted a survey of all respondents that were still in the sample villages when the children were 22 to 30 months old. In this study, we refer to this as the first follow-up survey. At the time of the first follow up, our sample included 1489 caregivers and toddlers.

In 2017, the most recent follow-up survey was conducted—around two years after the first follow-up survey—when the sample children were aged 49–65 months. We call this the second follow-up survey. During the second follow-up survey, we tracked 1572 children from the baseline sample. Of the 1572 children tracked, 1113 (71%) lived in rural villages, 287 (18%) lived in towns, 162 (10%) lived in counties, and 10 (<1%) lived in big cities, meaning that 29% of our sample had out-migrated from villages by the time of the second follow-up. In total, 1170 children were included in our final study sample, which included all children (and their families) that were surveyed in all three waves. For information on attrition and balance in our sample, please see Appendix B.

### 2.3. Data Collection

The data used in this study were collected in three survey waves: a baseline survey conducted between April 2013 and October 2013, a first follow-up survey conducted between October 2014 and April 2015, and a second follow-up survey conducted in June 2017. In all survey waves, the research team collected data on hemoglobin concentrations and anthropometric measures for all sample children, as well as socioeconomic characteristics of caregivers, children and households. We also collected data on the developmental outcomes of each sample child at baseline and during the second follow-up survey.

#### 2.3.1. Hemoglobin Concentrations

During all survey waves (2013, 2015 and 2017), nurses from Xi’an Jiaotong Medical School collected data on hemoglobin (Hb) concentrations from all sample children. Hb concentrations were measured using a HemoCue Hb 201+ finger prick system (Hemocue, Inc, Ängelholm, Sweden), which can obtain accurate Hb concentrations through one single drop of capillary blood [40]. Following international standards, at altitudes lower than 1000 m above sea level, we define anemia as an Hb concentration value of less than 110 g/L for children under 5 years old, and 115 g/L for children 5 years or older. We also adjusted the Hb concentration values for sample children living at altitudes higher than 1000 m above sea level, as the oxygen saturation of blood decreases with increased altitude. The altitude adjustment is based on the following formula: Hb adjustment = −0.032 × (altitude × 0.0032808) + 0.022 × (altitude × 0.0032808)^2^ [41]).

#### 2.3.2. Anthropometric Measures

We collected data on anthropometric measures for each child during all survey waves. Nurses measured the length and weight of the sample children to a precision of 0.1 units, according to WHO recommendations [42]. Each child’s weight was measured using a weight scale while the child was wearing light clothing and no shoes. In the baseline and first follow-up surveys, each child’s recumbent length was measured while barefoot and bareheaded using a standard infant length scale. In the second follow-up survey, an erect body length was measured with the child standing upright against a standard height ruler.

Anthropometric measurements were converted into physical indicators using the 2006 WHO child growth standards [43]. These physical indicators are for length-for-age z-scores (LAZ), weight-for-age z-scores (WAZ), and weight-for-length z-scores (WLZ). Following WHO standards, children whose LAZ, WAZ or WLZ scores are lower than two standard deviations (SD) below the intentional mean are considered to be stunted, underweight or wasted, respectively [44].

#### 2.3.3. Socioeconomic Characteristics

Trained enumerators collected socioeconomic data about the caregiver, the child and the household from each sample household during each survey wave. Enumerators first identified the primary caregiver as the individual most responsible for the child’s daily care (typically the child’s mother or grandmother). The primary caregiver was then asked a series of questions about the characteristics of the child, including gender (1 = male; 0 = female), whether the child had siblings (1 = yes; 0 = no), and whether the child was ever breastfed (1 = yes; 0 = no). Enumerators also asked the caregiver about characteristics of the household, including maternal age (1 = older than 25 years; 0 = 25 years or younger), maternal education level (1 = more than 9 years; 0 = 9 years or lower), and the value of family assets. We calculated a family asset index for each household using polychoric principal components analysis based on whether the family possessed the following items: tap water, a flush toilet, a water heater, a washing machine, a computer, internet, a refrigerator, an air conditioner, a motorcycle or electric bicycle, and a car. We did so because recent studies suggest that using a family asset index as an indicator of family wealth is more reliable than using self-reported income [45]). Each child’s age in months, premature birth status and birth weight were obtained from the child’s birth certificate. Children born before 37 weeks of gestation are considered premature, and children who weighed less than 2500 g at birth are considered to have a low birth weight.

#### 2.3.4. Child Cognitive Outcomes

At the time of the baseline survey (in 2013), we assessed the development outcomes of sample children using the first edition of the Bayley Scales of Infant Development (BSID). The BSID is an international scaled test for infants and toddlers under 3 years of age [46]. The test was formally adapted to the Chinese language and environment in 1992 and scaled according to an urban Chinese sample [47].

The BSID was administered by trained testers who underwent a formal week-long training course including 2.5 days of field training. The test was administered individually to each child using a standardized set of toys and a detailed scoring sheet. Caregivers were present but were not allowed to assist children during the administration of the BSID. The BSID takes into consideration each child’s age in days, as well as whether he or she was born prematurely. These factors, as well as the child’s performance on a series of tasks, are used to establish a mental development index (MDI), which measures memory, habitation, problem solving, early number concepts, generalization, classification, vocalization and language skills [46]. We use the MDI from the baseline survey as a control variable in our subsequent analysis.

In the second follow-up survey (in June 2017, when children were of preschool age), we measured child development outcomes using the Chinese version of the Wechsler Preschool and Primary Scale of Intelligence-Fourth Edition (WPPSI-IV) [48]. The WPPSI is an individually-administered, standardized, normative measure for assessing the cognitive functioning of children aged 4 to 6 years [48]. Extensive evidence has confirmed the structural validity and reliability of this test [48]. After revision, the Chinese version of the WPPSI-IV was formally adapted to the Chinese language and environment and has been applied in research across China [49,50,51].

As with the BSID, the WPPSI-IV was administered by trained enumerators who underwent a formal week-long training course, including 2.5 days of field training. The test was administered individually to each child, at either the child’s home or preschool, using a standardized set of toys and detailed scoring sheet. The WPPSI-IV protocol was strictly followed throughout the test. Neither caregivers nor teachers were allowed to assist the child during the administration of the WPPSI-IV.

The WPPSI-IV consists of five subtests that measure verbal comprehension, visual spatial, fluid reasoning, working memory, and processing speed. Taken together, these subtests produce a full-scale intelligence quotient (FSIQ), which is a composite score that summarizes cognitive ability across a diverse set of domains. This score is the most widely used measure of childhood intellectual function internationally [48].

### 2.4. Statistical Analysis

We sort sample children into five categories based on their dynamic anemia status over the three survey waves from infancy to preschool-age. These five categories are “persistently” anemic, “never” anemic, “deteriorating” anemia status, “improving” anemia status, and “fluctuating” anemia status. Children are considered to be persistently anemic if they were found to be anemic at baseline (in 2013) and at both follow-ups (in 2015 and 2017). Children in the never anemic group, in contrast, were not anemic at baseline or at either follow-up survey. If a child was not anemic at baseline and the first follow-up but had become anemic by the second follow-up, or if a child was not anemic at baseline but was found to be anemic at both the first and second follow-ups, we consider the child to have a deteriorating anemia status. If a child was anemic at baseline but was not anemic at both the first and second follow-up, or if a child was anemic at baseline and the first follow-up but was no longer anemic by the second follow-up, the child is considered to have an improving anemia status. Finally, children who fluctuated between anemic and non-anemic through the three survey waves (e.g., anemic at baseline, not anemic at the first follow-up, but anemic at the second follow-up) were included in the “fluctuating” group. We also sorted children into one of four categories (persistently anemic, never anemic, deteriorating anemia status, and improving anemia status) based on their dynamic anemia status from infancy to toddlerhood (baseline to first follow-up) and from toddlerhood to preschool-age (first follow-up to second follow-up), respectively.

For our analysis, WPPSI-IV cognitive raw scores (the sum of a child’s scores on the six subtests that contribute to FSIQ) are standardized. Since raw scores increase with age, we computed age-adjusted standardized cognitive scores by subtracting age-specific means and dividing by age-specific standard deviations (SDs) estimated using non-parametric regression methods. This non-parametric standardization method is less sensitive to outliers and small sample sizes within age groups and yields normally distributed standardized scores with a mean of zero across the age range (in months) [52].

To evaluate the cognitive differences of children in different categories of dynamic anemia status (both from infancy to toddlerhood and from toddlerhood to preschool age), we constructed a model as follows:

(1) $$Developmental\;Outcomes_{i}\;={\;\beta }_{0}+{\;\beta }_{1}Anemia\;Status_{i}+\;X_{i}\theta +\;u_{i}$$

where *Developmental Outcomes_i_* represents the FSIQ score of child *i* at preschool age (49–65 months old). When we compare the cognitive differences between never anemic children and children with deteriorating anemia status, the independent variable, *Anemia Status_i_*, is a dummy variable for the anemia status of child *i* that equals 1 for “deteriorating” and 0 for “never.” When we compare the cognitive differences between persistently anemic children and children with improving anemia status, *Anemia Status_i_* equals 1 for “persistent” and 0 for “improving.” We also compare the cognitive differences between never anemic and persistently anemic children, in which *Anemia Status_i_* equals 1 for “persistent” and 0 for “never.” The term *X_i_* is a vector of covariates that are included to capture the individual characteristics of each child (age, gender, whether he/she has siblings, whether he/she had low birth weight, whether he/she was ever breastfed, and MDI score at baseline), as well as the characteristics of each household (identity of primary caregiver, maternal age, maternal educational level, and family asset index). *u_i_* is an error term. We also control for county fixed effects.

In order to identify which child and household characteristics are most highly associated with each category of dynamic anemia status, we constructed a multivariate probit regression model as follows:

(2) $$Anemia\;Status_{i}\;={\;\beta }_{0}+{\;\beta }_{1}Child_{i}+{\;\beta }_{2}Household_{i}\;+\;u_{i}$$

When we compare the differences in characteristics between never anemic children and children with deteriorating anemia status, *Anemia Status_i_* equals 1 for “deteriorating” and 0 for “never.” When we compare the differences in characteristics between persistently anemic children and children with improving anemia status, *Anemia Status_i_* equals 1 for “persistent” and 0 for “improving.” *Child_i_* is a series of variables that capture an individual child’s characteristics, including gender, whether the child has siblings, whether the child had low birth weight, and whether the child was ever breastfed. *Household_i_* is a series of variables that capture household characteristics, including the identity of the primary caregiver, maternal age, maternal educational level, and family asset index. *u_i_* is a mean-zero error component, which captures unobserved factors that determine the dependent variable. We also control for county fixed effects and time fixed effects.

The analysis was conducted using STATA 14.2 (StataCorp, College Station, TX, USA). *p*-values below 0.05 are considered statistically significant.

## 3. Results

### 3.1. Socioeconomic and Demographic Characteristics of Participants

Table 1 presents the basic socioeconomic and demographic characteristics of study participants. Among the children in our sample, slightly more than half (51%) were male, and 75% did not have siblings. Only a small number of children in our sample (5%) had low birth weight. For 62% of sample children, the mother was the primary caregiver, while 35% of primary caregivers were paternal or maternal grandmothers, and only 3% of sample children were in the charge of other caregivers, such as fathers, grandfathers, aunts and uncles. Among mothers in our sample, around half (51%) were over 25 years of age. The majority of sample mothers (85%) had completed 9 years of schooling or less, which means only 15% of sample mothers had attained any high school education. The mean of family asset index was −0.07, which is slightly higher than that found in previous studies of western rural areas in China (−0.19) [21]. 

### 3.2. Hb Concentrations, Anemia, and Physical Development

The Hb concentrations, anemia status and physical development outcomes of sample children are reported in Table 2. When children were in infancy (6–12 months), the mean Hb concentration was 108.7 g/L, and slightly more than half of sample children (51%) were anemic (where anemia is defined as having an Hb concentration <110 g/L). As children aged, Hb concentrations increased, while the prevalence of anemia showed a decreasing trend. When children were in toddlerhood (22–30 months old), Hb concentrations were normally distributed with a mean of 118.1 g/L, and 24% of sample children were anemic. By the time sample children reached preschool-age (49–65 months), the average Hb concentration was 119.4 g/L and the anemia rate had decreased to 19%.

Looking next at physical development outcomes, indicators measured by LAZ, WAZ and WLZ show that the proportions of sample children who were stunted, underweight or wasting were much smaller than the proportion of children with anemia throughout all time periods. Rates of stunting, being underweight and wasting during infancy were 4%, 1% and 2%, respectively. In toddlerhood, rates of stunting decreased to 3%, while rates of being underweight and wasting showed no change. By the time the sample children reached preschool-age, rates of stunting and being underweight showed no change, while the rate of wasting was slightly higher (4%) than at toddlerhood (2%) (*p* < 0.01). However, even after this increase, the rates of wasting, stunting and being underweight were still significantly lower than the rate of anemia among sample children.

**Figure 1:**
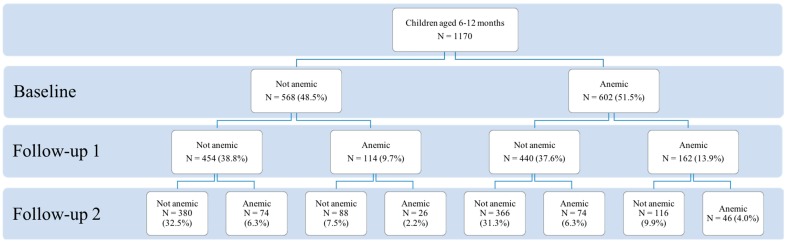
Crossover between normal and anemic states during the three waves of data collection. Data source: Author’s survey.

Figure 1 displays the prevalence of anemia over the course of the three survey waves. When we look at all sample children across the full age range of the study (6–65 months), we find that two thirds (67%) of sample children suffered from anemia at some point in time. This is higher than the prevalence of anemia in infancy (51%), toddlerhood (24%), or preschool age (19%). This means that in the long term, anemia affected a greater proportion of children than were affected in any one period from infancy to preschool-age. In the two follow-up survey waves (in toddlerhood and at preschool age), over a third (36%) of sample children were found to be anemic in at least one survey wave.

### 3.3. Dynamic Anemia Status

Table 3 presents the shares of sample children in each of the five categories of dynamic anemia status from infancy to preschool age. In our full sample of 1170 children, 380 children (33%) were never anemic, meaning that they did not suffer from anemia at any point of time from infancy to preschool-age. In the same period, 46 children (4%) were persistently anemic, meaning they were anemic in all three survey waves from infancy to preschool age. The data also show that 100 children (8%) had a deteriorating anemia status between infancy and preschool age. Of these 100 children, 26 developed anemia between infancy and toddlerhood, while 74 developed anemia between toddlerhood and preschool age. At the same time, 482 children (41%) had an improving anemia status. Among this group, 366 children saw their anemia status improve between infancy and toddlerhood, while 116 improved between toddlerhood and preschool age. Additionally, 162 sample children (14%) were included in the “fluctuating” anemia status group, indicating that they were inconsistently anemic between infancy and preschool-age.

Table A5 and Table A6 in Appendix C report the shares of children in each of the four categories of dynamic anemia status (persistently anemic, never anemic, improving anemia status, deteriorating anemia status), from infancy to toddlerhood and from toddlerhood to preschool-age, respectively. In the period between infancy and toddlerhood (Table A2), we find that 454 children (39%) of children were never anemic, and 162 children (14%) were persistently anemic. Additionally, 114 children (10%) had a deteriorating anemia status between infancy and toddlerhood, while 440 children (38%) saw their anemia status improve. From toddlerhood to preschool age (Table A3), 746 children (64%) were never anemic and 72 (6%) were persistently anemic. In the same period, 148 children (13%) saw their anemia status deteriorate, while 204 (17%) improved.

### 3.4. Dynamic Anemia Status and Child Cognitive Outcomes

Table 4 presents the correlations between categories of dynamic anemia status and preschool-age cognitive development. We find that children with improving anemia status score significantly higher in preschool-age standardized full-scale IQ (FSIQ) relative to children who were persistently anemic. Between infancy and preschool-age, children with improving anemia status scored 0.36 SD higher than persistently anemic children (*p* < 0.05). Improving children also scored 0.22 SD higher than persistently anemic children between infancy and toddlerhood (*p* < 0.01) and 0.29 SD higher between toddlerhood and preschool-age (*p* < 0.05).

The data also demonstrate that children who were persistently anemic have significantly lower scores relative to never anemic children. Specifically, standardized FSIQ scores for persistently anemic children were 0.33 SD lower from infancy to preschool-age than those of children who were never anemic (*p* < 0.05). Children with persistent anemia also scored 0.23 SD lower than never anemic children from infancy to toddlerhood (*p* < 0.01), and 0.31 SD lower from toddlerhood to preschool-age (*p* < 0.01). We find no significant differences in standardized FSIQ scores at any point in time between children with deteriorating anemia status and children who were never anemic.

### 3.5. Risk Factors of Dynamic Anemia Status

Table 5 presents the results of our multivariate probit analysis examining the associations between child and household characteristics and dynamic anemia status. Columns 1–2 specifically examine the characteristics correlated with deteriorating anemia status, while Columns 3–4 examine the correlates of improving anemia status. We find that from infancy to preschool-age, a deteriorating anemia status is positively correlated with low birth weight and negatively correlated with breastfeeding. Specifically, children with low birth weights were 11% more likely to have a deteriorating anemia status (rather than never being anemic) compared to children with normal birth weights (*p* < 0.01, column 2). Additionally, if a child was ever breastfed, he/she was 6% less likely to have a deteriorating status than a child who was never breastfed (*p* < 0.01, column 2). We also find that one factor is significantly correlated with improving anemia status from infancy to preschool-age: children with siblings were 5% more likely to have improving anemia status (rather than experience persistent anemia) compared to children without siblings (*p* < 0.01, column 8). However, we found no relationships between any household characteristics and any category of dynamic anemia status from infancy to preschool-age.

## 4. Discussion

This paper studies the dynamic anemia status of children from infancy to preschool age in rural northwestern China. Drawing on longitudinal data from 1170 children and their families in 11 rural counties in northwestern China, we examine the hemoglobin values and anemia status of children when they were in infancy, toddlerhood and preschool-age. We then sort sample children into categories of dynamic anemia status (persistently anemic, never anemic, deteriorating anemia status, improving anemia status, and fluctuating anemia status) and examine the differences in cognitive outcomes between children in each category. We also study risk factors associated with each category of dynamic anemia status.

The results demonstrate that the prevalence of anemia is high among rural infants (51%) and toddlers (24%). Such findings are consistent with previous studies in rural China, which have similarly found high rates of anemia among children under age three [13,14,15,21]. As the children in our sample reached preschool age, hemoglobin concentrations gradually rose, and the prevalence of anemia decreased slightly to 19%. However, this rate is still significantly higher than that of children from high-income countries such as the United States (9%), Canada (9%), and Norway (12%) [53]. Moreover, when we examine the overall prevalence of anemia over the course of the study (Figure 1), the results show that 67% of sample children were anemic at some point from infancy to preschool-age. This is higher than the prevalence of anemia at any one point during infancy (51%), toddlerhood (24%) and preschool-age (19%).

In contrast to the high rates of anemia in our sample, rates of wasting, stunting, and being underweight were much lower throughout the study. At infancy, toddlerhood and preschool-age, rates of stunting, wasting and being underweight never exceeded 4%. These rates are much lower than most other developing countries, where the prevalence of stunting, wasting, and being underweight are as high as 30% [54,55,56]. Our findings indicate that micronutrient deficiencies, rather than malnutrition, are the main nutritional challenge facing China’s rural children.

The data also reveal that many children showed changes in anemia status over the course of the study. Although only 4% of children were persistently anemic through infancy, toddlerhood and preschool-age, 8% of sample children saw their anemia status deteriorate, meaning that they developed anemia as they aged from infancy to preschool. In contrast, 41% of sample children saw their anemia status improve (meaning that they recovered from anemia between infancy and preschool age). Additionally, as children aged from 1 to 5 years old, 14% of the sample children fluctuated between anemic and non-anemic statuses.

Moreover, when we focus on the dynamic anemia status from infancy to toddlerhood (Table A2) and from toddlerhood to preschool-age (Table A3), the results show that sample children experienced more improvement and less deterioration in their anemia status between infancy and toddlerhood than between toddlerhood and preschool-age. Between infancy and toddlerhood, 38% of sample children had an improving anemia status, while only 10% deteriorated. In contrast, only 17% of sample children saw their anemia improve between toddlerhood and preschool age, while 13% of children deteriorated. These results suggest that children who are anemic in toddlerhood are less likely to recover from anemia by the time they reach preschool age.

Why are children less likely to recover from anemia during and after toddlerhood than before toddlerhood? One possible reason is that China’s central government has implemented a nutrition program in rural areas to combat infant anemia. The program, which began in 2012, distributes multiple micronutrient powders (MNPs) to rural infants and toddlers aged 6–24 months, and, to date, the program has made huge progress in reducing anemia and improving the health status of targeted children [57]. However, the program ends when children reach 24 months, and there is no national nutrition program for children aged 2–5 years in rural China. Our results suggest that without nutritional intervention, rural toddlers and preschool-age children may be more likely to develop anemia and less likely to recover. Therefore, there is an urgent need for nutritional interventions to prevent and treat anemia among rural toddlers as they grow.

As predicted, we found that persistently anemic children have significantly lower cognitive scores relative to children who were never anemic. In fact, this fundamental finding is supported by the previous literature on childhood anemia and cognitive development, which has found that anemic children show lower levels of cognition than non-anemic children [4,5,6,7,8,9,10,11,12]. The results also demonstrate that, in addition to children who are never anemic, children who recover from anemia also have better developmental outcomes than persistently anemic children. One previous study has also produced a similar result—among infants in Chile, those whose anemia persisted for a longer period had significantly lower motor and cognitive scores compared with infants whose anemia persisted for a shorter period [26]. Taken together with previous research, the results of this study show that in order to prevent the negative effects of anemia on child cognitive development, anemia must be addressed as soon as possible after diagnosis.

Finally, we identified a relatively small number of individual characteristics that are able to predict improving or deteriorating anemia statuses from infancy to preschool-age. Specifically, according to the results, children who had been breastfed were less likely to have a deteriorating anemia status, and children with low birth weight were more likely to experience a deteriorating anemia status. Such findings, in fact, are consistent with previous international research, which has found that longer breastfeeding duration is correlated with higher hemoglobin concentrations [27], while low birth weight is linked to childhood anemia [34,35,36]. Additionally, the authors found that children who have siblings were more likely to have an improving anemia status, which is consistent with previous studies in China [58].

In contrast, the results find no associations between dynamic anemia status and any household socioeconomic characteristics, including maternal age, maternal educational level and family asset index. This finding is similar to the quantitative results of a mixed-methods study of children in rural China, which found no significant relationships between socioeconomic variables and the prevalence of anemia [21]. According to the qualitative results of the study, the reason for this lack of significant correlation is that, regardless of socioeconomic status, most rural caregivers have a poor understanding of anemia and of how to prevent or treat anemia in children [21]. This may also be true among this study’s sample, which would explain why deteriorating or improving anemia statuses are not associated with any socioeconomic variables.

These findings have clear implications for both policymakers and researchers. First, considering the high rates of improvement in anemia status between infancy and preschool, we recommend that China continues to support and expand its national MNP program. As of 2015, more than 2.67 million children in 341 nationally-designated poverty counties in rural China have benefited from the program [57]. However, millions of children are still not covered by the program and are at risk of micronutrient deficiency [57]. Therefore, the government should invest in providing full coverage of the program to all children aged 6–24 months in China’s poverty-stricken areas.

In addition, the results suggest that anemia in toddlerhood and preschool-age should receive greater attention from policymakers, as children show the most deterioration and least improvement during this period. Whereas children aged 6–24 months can receive micronutrients through the national MNP program, there are no resources to help children older than 24 months to combat anemia. We therefore recommend that the national government not only expand the MNP program geographically, but also invest in expanding the program to cover all rural children up to age five. This may also help to reduce the prevalence of cognitive delays among China’s rural children, which our results show to be positively linked to persistent childhood anemia and negatively linked to improving anemia status.

This study makes three key contributions to the literature. First, it is the first to investigate longitudinal changes in anemia status among children in developing regions, where childhood anemia is most prevalent. The study also is the first in a developing setting to examine correlates of changes in anemia status. Second, this is the only recent study to provide up-to-date information on the prevalence of anemia among preschool-aged children in rural China, as well as the true share of children affected by anemia in the period from infancy to preschool-age. Finally, this is the first study to examine the associations between dynamic anemia status and early childhood development.

We also acknowledge two limitations of this study. First, although we document changes in anemia status from infancy to preschool-age, the data were collected in three survey waves separated by intervals of nearly two years. As a result, this analysis may underestimate the true share of children who were affected by anemia through early childhood. Future studies should examine the changes in childhood anemia status over shorter intervals to better understand dynamic anemia status in both the short and long term. Additionally, we were unable to conduct full blood panel testing and thus cannot identify the specific type of anemia (e.g., megaloblastic, normocytic or microcytic) among the sample children. However, previous research reports that 85–95% of anemia in China is caused by iron deficiency [59].

## 5. Conclusions

In conclusion, the results demonstrate that anemia affects a large share of children in rural China. Although anemia rates appear to fall as children age, they remain high among preschool-age children. Additionally, the study’s findings suggest that children may be at greater risk for developing anemia, or for having persistent anemia, during the period between toddlerhood and preschool-age. Since children with improving anemia status have higher developmental scores than children with persistent anemia, there is an urgent need for effective strategies to prevent anemia and treat anemia as children grow, especially between toddlerhood and preschool age. Expanded nutritional interventions from infancy through preschool-age should therefore be implemented to combat childhood anemia and improve child development.

## Figures and Tables

**Table 1:** Basic characteristics of sample children (6–12 months) (n = 1170).

Characteristics	Frequency (n)	Percentage (%)/Mean ± SD
	(1)	(2)
***Child characteristics***		
Gender		
Male	599	51.2
Female	571	48.8
Has siblings		
Yes	289	24.7
No	881	75.3
Low birth weight		
Yes	61	5.2
No	1109	94.8
Was ever breastfed		
Yes	688	58.8
No	482	41.2
***Household characteristics***		
Mother is primary caregiver		
Yes	730	62.4
No	440	37.6
Maternal age		
Age ≤ 25	576	49.2
Age > 25	594	50.8
Maternal education level (years)		
≤9	995	85.0
>9	175	15.0
Family asset index	1170	−0.07 ± 1.17

Notes: Data source is the author’s survey. Descriptive statistics of child and household characteristics when children are 6–12 months of age. The two columns show the frequency and percentage of each characteristic, respectively. The asset index is constructed using polychoric principal components on the following variables: tape water, toilet, water heater, wash machine, computer, internet, fridge, air condition, motor or electronic bicycle, and car.

**Table 2:** Hb concentration, anemia and physical development.

Variables	Infancy	Toddlerhood	Preschool-Age	Differences (1)–(2)	Differences (1)–(3)	Differences (2)–(3)
	Mean/SD	Mean/SD	Mean/SD	*p*-Value	*p*-Value	*p*-Value
	(1)	(2)	(3)	(4)	(5)	(6)
Child’s hemoglobin value	108.7	118.1	119.4	0.000	0.000	0.010
(g/L)	(12.49)	(13.13)	(10.44)			
Anemic	0.51	0.24	0.19	0.000	0.000	0.005
	(0.50)	(0.42)	(0.39)			
Stunted	0.04	0.03	0.02	0.187	0.024	0.340
(LAZ ≤ −2)	(0.19)	(0.16)	(0.14)			
Underweight	0.01	0.01	0.02	0.344	0.043	0.274
(WAZ ≤ −2)	(0.10)	(0.12)	(0.14)			
Wasted	0.02	0.02	0.04	0.165	0.053	0.001
(WLZ ≤ −2)	(0.15)	(0.13)	(0.19)			
Observations	1170	1170	1170			

Notes: Data source is the author’s survey. We define anemia as an Hb concentration value of less than 110 g/L for children under 5 years old, and 115 g/L for children older than 5 years old. Data are presented as mean and SD in column 1–3. SD is shown in parentheses. Column 1–3 shows health outcomes, respectively, when children are in infancy (6–12 months), toddlerhood (22–30 months) and preschool-age (49–65 months). Column 4 presents the p-values of the differences between column 1 and column 2. Column 5 presents the p-values of the differences between column 1 and column 3. Column 6 presents the p-values of the differences between column 2 and column 3. LAZ means length-for-age Z score, WAZ means weight-for-age Z score, and WLZ means weight-for-length Z score.

**Table 3:** Dynamic anemia status from infancy to preschool-age.

Variables	Infancy	Toddlerhood	Preschool-Age	Frequency (n)	Percentage (%)
Never	NO	NO	NO	380	33
Persistent	YES	YES	YES	46	4
Deteriorating	NO	NO	YES	74	6
	NO	YES	YES	26	2
Improving	YES	NO	NO	366	31
	YES	YES	NO	116	10
Fluctuating	YES	NO	YES	74	6
	NO	YES	NO	88	8
**Observations**				**1170**	**100**

Notes: Data source is the author’s survey. Every sample child was assigned to one category based on his/her anemia status in infancy, toddlerhood and preschool-age. Dynamic anemia status is presented in both frequency and percentage.

**Table 4:** Differences cognitive outcomes between anemia status.

Variable	Standardized FSIQ (Preschool-Age)								
	Infancy to Preschool-Age			Infancy to Toddlerhood			Toddlerhood to Preschool-Age		
	(1)	(2)	(3)	(4)	(5)	(6)	(7)	(8)	(9)
Improving	0.36 **			0.22 ***			0.29 **		
(1 = Improving, 0 = Persistent)	(0.14)			(0.09)			(0.13)		
Deteriorating		0.02			-0.03			0.01	
(1 = Deteriorating, 0 = Never)		(0.11)			(0.10)			(0.08)	
Persistent			−0.33 **			−0.23 ***			−0.31 ***
(1 = Persistent, 0 = Never)			(0.15)			(0.09)			(0.12)
Baseline MDI scores	YES	YES	YES	YES	YES	YES	YES	YES	YES
Control variables	YES	YES	YES	YES	YES	YES	YES	YES	YES
County fixed effect	YES	YES	YES	YES	YES	YES	YES	YES	YES
Observations	528	480	426	602	568	616	276	894	818

Notes: Data source is the author’s survey. Control variables include the child’s age, gender, whether the child had low birth weight, whether the child has siblings, whether the child was ever breastfed, whether the age of the mother is above 25 years old, whether the mother of the child had attained more than 9 years of education, the primary caregiver of the child, and the family asset index. We also control for baseline Bayle mental development index (MDI) scores and county fixed effect. ** significant at the 5% level; *** significant at the 1% level. FSIQ: full-scale intelligence quotient.

**Table 5:** Multivariate analysis of the association between characteristics and anemia status.

Variable	Deteriorating (1 = Deteriorating, 0 = Never)		Improving (1 = Improving, 0 = Persistent)	
	Infancy to Preschool-Age		Infancy to Preschool-Age	
	β	ME	β	ME
	(1)	(2)	(3)	(4)
***Child’s characteristics***				
Male	0.13	0.03	−0.10	−0.02
(1 = yes)	(0.08)	(0.02)	(0.10)	(0.01)
Has siblings	0.12	0.03	0.35 ***	0.05 ***
(1 = yes)	(0.09)	(0.02)	(0.12)	(0.02)
Low birth weight	0.42 ***	0.11 ***	0.02	0.00
(1 = yes)	(0.16)	(0.04)	(0.21)	(0.03)
Was ever breastfed	−0.24 ***	−0.06 ***	0.16	0.02
(1 = yes)	(0.09)	(0.02)	(0.11)	(0.02)
***Household characteristics***				
Primary caregiver	−0.05	−0.01	0.00	0.00
(1 = mother)	(0.09)	(0.02)	(0.12)	(0.02)
Maternal age	0.04	0.01	0.08	0.01
(1 = above 25 years old)	(0.08)	(0.02)	(0.10)	(0.01)
Maternal education level	−0.07	−0.02	0.04	0.01
(1 = 9 years or higher)	(0.11)	(0.03)	(0.14)	(0.02)
Family asset index	0.03	0.01	0.08	0.01
	(0.04)	(0.01)	(0.04)	(0.01)
County fixed effect	YES	YES	YES	YES
Time fixed effect	YES	YES	YES	YES
Observations	1440	1440	1584	1584

Notes: Data source is the author’s survey. Column 1 shows coefficients and standard errors (in parentheses) from probit regression. Column 2 shows marginal effects from the same probit regression where the dependent variable represents 1 for “deteriorating” and 0 for “never” when the child is aged from infancy (6–12 months) to preschool-age (49–65 months). The same multivariate analysis is conducted in “improving” and “persistent” groups, which are shown in columns 3–4. The dependent variable represents 1 for “improving” and 0 for “persistent”. All regressions control for the county fixed effect and time fixed effect. *** indicates significance at 1%.

**Figure A1:**
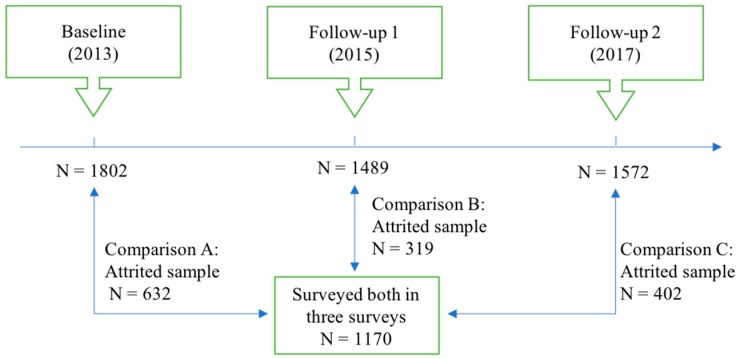
Sample size of each survey round.

## Appendix A

In this section, we examine two additional factors that may also be linked to the dynamic anemia status among the sample children: dietary diversity and maternal anemia status. We fist review the literature on dietary diversity and maternal anemia in relation to the anemia status of children. Next, we describe the data collected in this study. Finally, we examine the correlations between the dietary diversity, maternal anemia status, and dynamic anemia status among the children in our sample.

The international literature has shown that diet and maternal anemia status are both closely related to anemia in children. Research has found that both the quantity and quality of a child’s diet is linked to anemia in a variety of contexts [60,61]. For example, a study of children under two years in Ethiopia found that children with less diverse diets were significantly more likely to be anemic than children who ate a greater diversity of foods [60]. Additionally, a wide array of literature has found that maternal anemia status can predict the anemia status of children [33,62,63]. Studies conducted in Indonesia and Mexico have both found that maternal anemia is significantly associated with childhood anemia [33,63]. Additionally, a study conducted in poor areas of rural China in 2010 found that, for children aged 0–11 months, maternal anemia is positively correlated with childhood anemia [62].

Based on this literature, it is reasonable to consider whether maternal anemia may be linked to dynamic anemia status among children in our sample. In fact, we collected information on both child dietary diversity and maternal anemia status during this study. We collected dietary diversity information of half of the sample children at baseline (infancy) and all children in the sample in the first and second follow-up surveys. Following the recommendations of the World Health Organization [64], we provided caregivers with a list of seven food types of food and asked caregivers to identify which food types their child had consumed yesterday (the seven food types include (1) grains, roots, and tubers; (2) legumes and nuts; (3) dairy products; (4) flesh foods (meat, fish, poultry, and liver/organ meats); (5) eggs; (6) vitamin-A-rich fruits and vegetables; and (7) other fruits and vegetables). We then calculated dietary diversity scores by summing the number of food types that caregivers identified. We also measured maternal Hb concentrations during the baseline survey wave using a HemoCue Hb 201+ finger prick system (Hemocue, Inc, Ängelholm, Sweden). Anemia is defined as Hb < 120 g/L for non-pregnant women above 15 years old and as Hb < 110 g/L for pregnant women [65]. We then regressed child dietary diversity scores and maternal anemia status against changes in anemia status using Equation (2).

Table A1 shows the dietary diversity and maternal anemia status among our sample. The average dietary diversity score across the three survey waves is 4.03 out of a possible seven. Sample children scored 2.63 out seven in dietary diversity during infancy, 4.26 during toddlerhood and 4.42 at preschool age. Mothers in our sample had Hb concentrations of 127.7 g/L, and 21% of mothers of sample children were anemic at baseline.

**Table A1:** Descriptive statistics for the dietary diversity and maternal anemia status of sample children.

Characteristics.	Percentage (%)/Mean ± SD
Dietary diversity	4.03 ± 1.56
Infancy	2.63 ± 1.57
Toddlerhood	4.26 ± 1.31
Preschool-age	4.42 ± 1.44
Maternal hemoglobin values (g/L)	127.7 ± 13.5
Maternal anemia at baseline	20.9

Table A2 presents the correlations between dietary diversity and dynamic anemia status. We find that childhood dietary diversity is not significantly associated with deteriorating or improving anemia status in any time period among our sample. This contrasts with previous literature, which has found a close relationship between a child’s diet and anemia status [60,61]. A review of the literature suggests two possible reasons for the results. First, our survey only assessed how many food types each sample child consumed the previous day; we did not examine portion sizes or the diversity of foods within each food type. The second possible explanation is that the consumption of iron-rich foods was generally low in our study sample, which means that the statistical power may be too low to detect significant associations. Other studies also have reported a similar lack of association between dietary diversity and anemia due to these reasons [66,67]. The exact reason and mechanisms behind this finding are beyond the scope of our study, and future research is needed to further investigate the relationship between food intake and anemia among children in rural China.

**Table A2:** Multivariate analysis of the association between dietary diversity and evolutionary paths.

Variable	Deteriorating (1 = Deteriorating, 0 = Never)		Improving (1 = Improving, 0 = Persistent)	
	Infancy to Preschool-Age		Infancy to Preschool-Age	
	β	ME	β	ME
	(1)	(2)	(3)	(4)
Dietary diversity scores	0.00	0.00	−0.03	−0.00
	(0.03)	(0.01)	(0.04)	(0.01)
Control variables	YES	YES	YES	YES
County fixed effect	YES	YES	YES	YES
Time fixed effect	YES	YES	YES	YES
Observations	1214	1214	1256	1256

Note: Data source is the author’s survey. Control variables include a child’s age, gender, whether the child had low birth weight, whether the child has siblings, whether the child was ever breastfed, whether the age of the mother is above 25 years old, whether the mother of the child had attained more than 9 years of education, the primary caregiver of the child, and the family asset index.

Table A3 presents the correlations between maternal anemia status and dynamic anemia status among the sample children. In contrast to diet, we find that maternal anemia is significantly correlated with deteriorating anemia status in children. Specifically, if a child’s mother was anemic at baseline, that child was 9% more likely to experience a deteriorating anemia status between infancy and preschool (*p* < 0.01, column 2). This is consistent with much of the literature, which has found links between maternal anemia and childhood anemia [33,62,63]. However, we find no significant relationship between maternal anemia status and improving anemia status among children. Considering the prevalence of maternal anemia among our sample (21%), these results suggest that policy efforts should also aim to improve the micronutrient intake of rural mothers, in addition to children.

**Table A3:** Multivariate analysis of the association between maternal anemia status and evolutionary paths.

Variable	Deteriorating (1 = Deteriorating, 0 = Never)		Improving (1 = Improving, 0 = Persistent)	
	Infancy to Preschool-Age		Infancy to Preschool-Age	
	β	ME	β	ME
	(1)	(2)	(3)	(4)
Maternal anemia	0.34 ***	0.09 ***	−0.07	−0.01
(1 = yes)	(0.10)	(0.03)	(0.12)	(0.02)
Control variables	YES	YES	YES	YES
County fixed effect	YES	YES	YES	YES
Time fixed effect	YES	YES	YES	YES
Observations	1209	1209	1266	1266

Note: Data source is the author’s survey. Control variables include the child’s age, gender, whether the child had low birth weight, whether the child has siblings, whether the child was ever breastfed, whether the age of the mother is above 25 years old, whether the mother of the child had attained more than 9 years of education, the primary caregiver of the child, and the family asset index. *** indicates significance at 1%.

## Appendix B

Our original sample included 1802 infants aged 6–11 months. We followed up with 1489 children when they were 22–30 months old (in 2015, at the first follow-up for this study), and 1572 children when they were 49–65 months old (in 2017, at the second follow-up survey for this study). Our final sample included 1170 children and their caregivers, all of whom were present for all three survey waves (Figure A1). The attrition rate was 17.4% at the first follow-up survey, and 12.8% at the second follow-up survey. Overall, the attrition rates were low.

Table A4 compares the attrited sample and the non-attrited sample at three points in time. Comparison A (columns 1–4) compares the final sample (n = 1170) with the sample that attrited between the baseline and the final sample (n = 632). The data show that children in the attrition group were less likely to be anemic (*p* < 0.01). However, although the hemoglobin values of attrited children were higher than the non-attrited children (*p* = 0.015), the magnitude of this difference was small (1.53 g/L). In addition, compared with non-attrited children, children in the attrition group were more likely to be the only child in their family (*p* < 0.01), and they were more likely to be cared for by family members other than mothers (*p* < 0.01; in our sample, this was usually the grandmother). Children in the attrition group also tended to have mothers with higher education levels.

Comparison B (columns 5–8) compares children in the final sample (n = 1170) with the sample of children who attrited between the first follow-up and the final sample (n = 319). The data show that attrited children were more likely to have no siblings (*p* < 0.01) and mothers were less likely to be primary caregivers in the attrited sample (*p* < 0.01). However, there are no statistically significant differences in health outcomes or anemia status between attrited and non-attrited children.

The same results can be found when we compare the final sample (n = 1170) to the sample of children who attrited between the second follow-up and the final sample (n = 402) (Comparison C, columns 9–12). Attrited children were more likely to have no siblings (*p* < 0.01) and mothers were less likely to be primary caregivers in the attrited sample (*p* < 0.01). Again, no difference can be found in child health outcomes between attrited and non-attrited children.

Overall, we can see that children in the attrition group tend to be only children and to be cared for by grandmothers rather than mothers. Although attrited children were more likely to be taken care of by grandmothers compared with non-attrited samples—which could be negatively associated with a child’s development outcomes [68]—mothers also count for the larger share of primary caregivers in our sample, both before and after attrition. Mothers of attrited children also tend to be more educated, indicating a slightly higher socioeconomic status. This means that our final sample may be more vulnerable in some ways (such as parental investment) and less vulnerable in other ways (such as socioeconomic status). It is also important to note that we control for all child and household characteristics in our multivariate analysis. Therefore, it is unlikely that attrition impacted the results of this study.

**Table A4:** Summary statistics of outcomes by attrition status.

	Comparison A				Comparison B				Comparison C			
	Baseline (Infancy)				Follow-Up 1 (Toddlerhood)				Follow-Up 2 (Preschool-Age)			
	Full Sample	Attrited Sample	Non-Attrited Sample	Differences (2)–(3)	Full Sample	Attrited Sample	Non-Attrited Sample	Differences (6)–(7)	Full Sample	Attrited Sample	Non-Attrited Sample	Differences (10)–(11)
	Mean/SD	Mean/SD	Mean/SD	*p*-Value	Mean/SD	Mean/SD	Mean/SD	*p*-Value	Mean/SD	Mean/SD	Mean/SD	*p*-Value
	(1)	(2)	(3)	(4)	(5)	(6)	(7)	(8)	(9)	(10)	(11)	(12)
***Health outcomes***												
Child’s hemoglobin value	109.19	110.19	108.66	0.015	118.37	119.60	118.12	0.105	119.58	120.31	119.39	0.165
(g/L)	(12.58)	(12.69)	(12.49)		(13.07)	(12.73)	(13.13)		(10.49)	(10.66)	(10.44)	
Anemic	0.49	0.43	0.51	<0.001	0.23	0.20	0.24	0.214	0.19	0.18	0.19	0.721
(hb < 110 g/L)	(0.50)	(0.50)	(0.50)		(0.42)	(0.40)	(0.42)		(0.39)	(0.38)	(0.39)	
Stunted	0.04	0.03	0.04	0.822	0.03	0.03	0.03	0.685	0.02	0.03	0.02	0.149
(LAZ ≤ −2)	(0.18)	(0.18)	(0.19)		(0.16)	(0.17)	(0.16)		(0.15)	(0.18)	(0.14)	
Underweight	0.01	0.01	0.01	0.846	0.01	0.01	0.01	0.292	0.02	0.02	0.02	0.673
(WAZ ≤ −2)	(0.10)	(0.11)	(0.10)		(0.11)	(0.08)	(0.12)		(0.14)	(0.13)	(0.14)	
Wasted	0.02	0.02	0.02	0.672	0.02	0.01	0.02	0.446	0.04	0.03	0.04	0.532
(WLZ ≤ −2)	(0.15)	(0.14)	(0.15)		(0.12)	(0.10)	(0.13)		(0.19)	(0.17)	(0.19)	
***Child’s characteristics***												
Child’s age	9.50	9.59	9.44	0.100	27.47	27.60	27.44	0.145	56.86	56.84	56.87	0.903
(in months)	(1.83)	(1.88)	(1.81)		(1.77)	(1.79)	(1.76)		(3.42)	(3.52)	(3.38)	
Male	0.53	0.56	0.51	0.059	0.52	0.55	0.51	0.208	0.52	0.55	0.51	0.163
(1 = yes)	(0.50)	(0.50)	(0.50)		(0.50)	(0.50)	(0.50)		(0.50)	(0.50)	(0.50)	
Have siblings	0.21	0.13	0.25	<0.001	0.22	0.13	0.25	<0.001	0.45	0.38	0.47	0.002
(1 = yes)	(0.41)	(0.34)	(0.43)		(0.42)	(0.34)	(0.43)		(0.50)	(0.49)	(0.50)	
Low Birth Weight	0.05	0.04	0.05	0.199	0.05	0.03	0.05	0.215	0.05	0.04	0.05	0.258
(1 = yes)	(0.21)	(0.19)	(0.22)		(0.22)	(0.18)	(0.22)		(0.22)	(0.19)	(0.22)	
Was ever breastfed	0.58	0.57	0.59	0.412	0.58	0.53	0.59	0.062	0.59	0.59	0.59	0.888
(1 = yes)	(0.49)	(0.50)	(0.49)		(0.49)	(0.50)	(0.49)		(0.49)	(0.49)	(0.49)	
***Household characteristics***												
Primary caregiver	0.82	0.78	0.85	<0.001	0.60	0.52	0.62	<0.001	0.61	0.54	0.63	0.003
(1 = mother)	(0.38)	(0.41)	(0.36)		(0.49)	(0.50)	(0.48)		(0.49)	(0.50)	(0.48)	
Maternal age	0.50	0.48	0.51	0.280	0.51	0.52	0.51	0.688	0.50	0.49	0.51	0.434
(1 = above 25 years old)	(0.50)	(0.50)	(0.50)		(0.50)	(0.50)	(0.50)		(0.50)	(0.50)	(0.50)	
Maternal education level	0.17	0.21	0.15	0.003	0.16	0.18	0.15	0.194	0.16	0.18	0.15	0.165
(1 = 9 years or higher)	(0.37)	(0.40)	(0.36)		(0.36)	(0.39)	(0.36)		(0.36)	(0.38)	(0.36)	
Family asset index	−0.04	0.02	−0.07	0.091	−0.04	0.06	−0.07	0.070	−0.35	−0.32	−0.36	0.557
	(1.18)	(1.20)	(1.17)		(1.19)	(1.24)	(1.17)		(1.19)	(1.14)	(1.20)	
Observations	1802	632	1170		1489	319	1170		1572	402	1170	

Notes: Data source is authors’ survey.

## Appendix C

**Table A5:** Evolutionary path from infancy to toddlerhood.

Variables	Infancy	Toddlerhood	Frequency (n)	Percentage (%)
Never	NO	NO	454	39
Persistent	YES	YES	162	14
Deteriorating	NO	YES	114	10
Improving	YES	NO	440	38
**Observations**			**1170**	**100**

**Table A6:** Evolutionary path from toddlerhood to preschool-age.

Variables	Toddlerhood	Preschool-Age	Frequency (n)	Percentage (%)
Never	NO	NO	746	64
Persistent	YES	YES	72	6
Deteriorating	NO	YES	148	13
Improving	YES	NO	204	17
**Observations**			**1170**	**100**
